# First record of the complete chloroplast genome of *Syneilesis aconitifolia* (Asteraceae)

**DOI:** 10.1080/23802359.2022.2126734

**Published:** 2022-10-12

**Authors:** Sang-Chul Kim, Young-Ho Ha, Kyung Choi, Hyuk-Jin Kim

**Affiliations:** Division of Forest Biodiversity, Korea National Arboretum, Pocheon-si, Gyeonggi-do, Korea

**Keywords:** *Syneilesis aconitifolia*, Asteraceae, Senecioneae, chloroplast genome, phylogenetic

## Abstract

*Syneilesis aconitifolia* is an herbaceous perennial of the Asteraceae family native to forests in China, Korea, Japan, and eastern Russia. In Korea, the young leaves of the plant are edible and the extract is known to have antitumor effects. The length of the complete plastome was found to be 150,773 bp, including 130 genes, consisting of 85 protein-coding genes, 37 tRNA genes, and 8 rRNA genes. The assembled plastome showed typical structure and gene content of the angiosperm plastome, which includes two inverted repeats (IR) regions of 24839 bp, a large single copy (LSC) region of 82911 bp, and a small single-copy (SSC) region of 18184 bp. The total G/C content in the *S. aconitifolia* plastome was 37.5%. The maximum likelihood (ML) phylogenetic tree strongly supports that *S. aconitifolia* is closely related to the hosts of *Ligularia fischeri*. This study reports the first complete chloroplast genome of the genus *Syneilesis* and will contribute to the phylogenetics of the family Asteraceae.

## Introduction

*Syneilesis aconitifolia* (Bunge) Maxim. 1859 (Maximowicz 1859), commonly known as the shredded umbrella plant, is an herbaceous perennial of the Asteraceae family that is native to forests in China, Korea, Japan, and eastern Russia. In Korea, the young leaves of the plant are edible and the extract is known to have antitumor effects (Wu et al. 2011). The genus *Syneilesis* contains seven species in the world, which are *Syneilesis aconitifolia, Syneilesis subglabrata, Syneilesis australis, Syneilesis hayatae, Syneilesis akagii, Syneilesis palmata* and *Syneilesis tagawae*, and complete chloroplast genome has not yet been reported. Here, we report for the first time the complete sequence of the chloroplast genome of *S. aconitifolia*, and propose a molecular phylogenetic relationship in relation to the chloroplasts of other plants in the Senecioneae of Asteraceae.

*S. aconitifolia* plant material was collected from Haeri-myeon, Gochang-gun, Jeollabuk-do, Republic of Korea (35°27′ 43.45″ N, 126°33'11.14″ E). Total genomic DNA was extracted using the DNeasy Plant Mini Kit (Qiagen Inc., Valencia, CA, USA), according to the manufacturer’s instructions. The extracted DNA was quantified using the NanoDrop 2000 (Thermo Fisher Inc., Waltham, MA, USA), and the quantity was confirmed using a 1% agarose gel. Voucher specimens of *S. aconitifolia* accessions were deposited in the herbarium of the Korea National Arboretum (http://www.nature.go.kr/kbi/plant/smpl/KBI_2001_030100.do, Hee Young Gil, E-mail: warmishe@korea.kr, Voucher number: ESK21-613). Illumina paired-end libraries were constructed and sequenced on the MiSeq platform (Macrogen Inc., Seoul, South Korea). The chloroplast sequence data were filtered and generated using GetOrganelle (Jin et al. 2018). Finally, the chloroplast (cp) cp genome was assembled using Geneious Prime (Kearse et al. 2012) and annotated using GeSeq (Tillich et al. 2017). Unannotated portions such as exons and introns were manually edited. The tRNA sequences were confirmed using tRNAscan-SE 1.21 (Lowe and Chan 2016).

The complete chloroplast genome of *S. aconitifolia* was found to be a circular DNA molecule of 150,773 bp in length, separated into a large single-copy region of 82,911 bp, a small single-copy region of 18,184 bp, and a pair of inverted repeats (IRa and IRb) of 24,839 bp. The genome contained 130 genes, including 85 protein-coding genes, 37 tRNA genes, and eight rRNA genes, and showed a GC content of 37.5% (LSC: 35.6%, SSC: 30.8%, IRs: 43.0%). Among the identified genes, six protein-coding genes, seven tRNA genes, and four rRNA genes were completely duplicated in the IR regions. Eight tRNA genes and ten protein-coding genes contained one intron, and two protein-coding genes (*clp*P1 and *paf*I) contained two introns. The complete chloroplast genome of *S. aconitifolia* was submitted to GenBank with the accession number OM622255, which is the first reported chloroplast genome sequence in the genus *Syneilesis*.

Phylogenetic analysis of *S. aconitifolia* was performed by comparing with 79 protein-coding gene sequences derived from the chloroplast genome sequences of other 11 species in the family Asteraceae and two outgroups (family Menyanthaceae). 79 protein-coding gene sequences were aligned using MAFFT in PhyloSuite (Katoh et al. 2005; Zhang et al. 2020), and ModelFinder (Kalyaanamoorthy et al. 2017) within the PhyloSuite program was used to determine the optimal alternative model. ML analysis was performed using IQ-tree and TVM + F+R3 models (Nguyen et al. 2015). *S. aconitifolia* was a sister group of the genus *Ligularia.* In addition, within Senecioneae, Tussilagininae and Senecioninae were distinguished.

**Figure 1.**
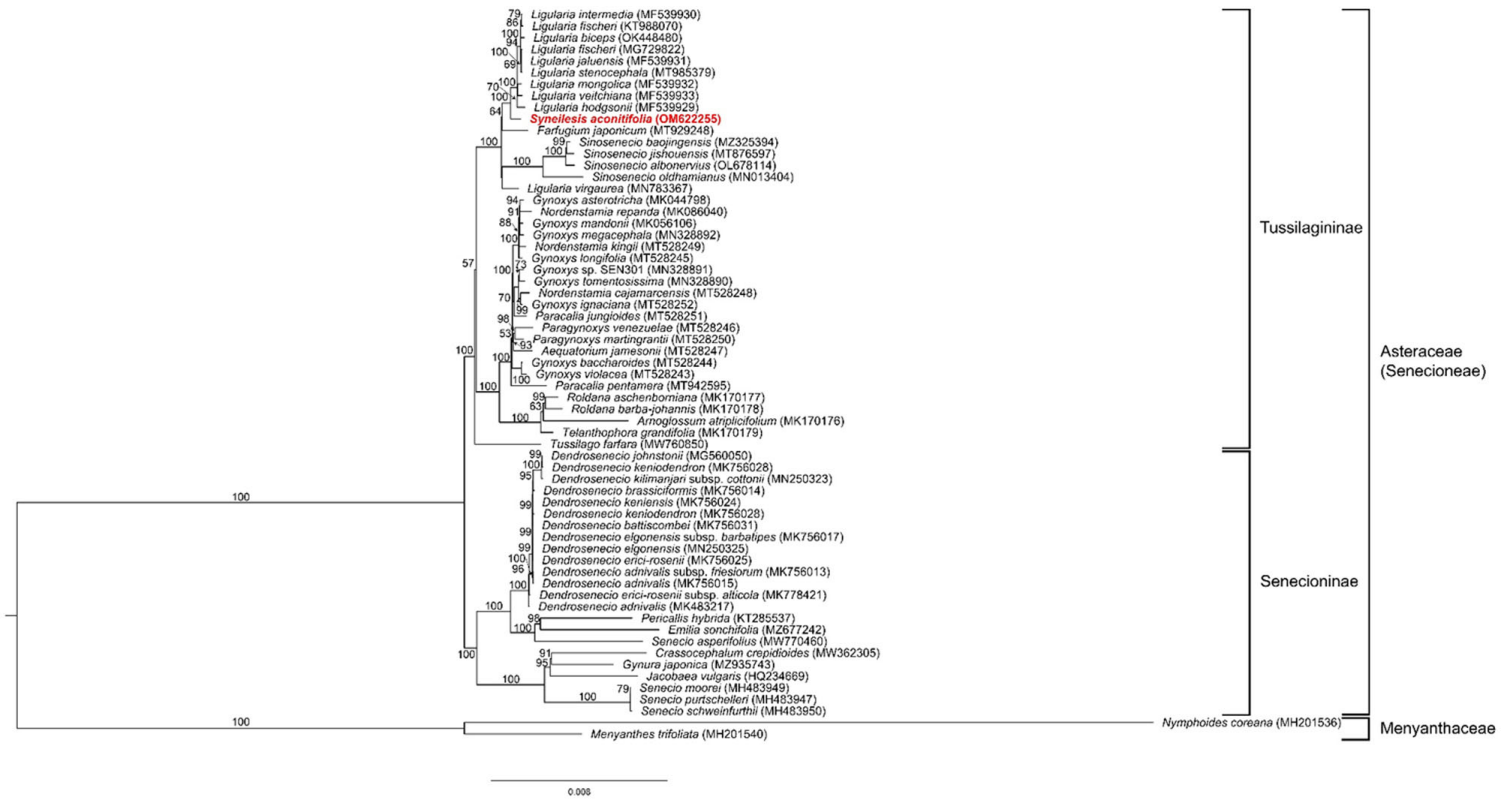
Maximum likelihood phylogenetic tree of *S. aconitifolia* with other 63 species based on 79 protein-coding gene sequences. Numbers in the nodes represent bootstrap values from 50,000 replicates.

## Data Availability

The genome sequence data that support the findings of this study are openly available in GenBank of NCBI at [https://www.ncbi.nlm.nih.gov] (https://www.ncbi.nlm.nih.gov/) under accession no. OM622255. The associated BioProject, SRA, and Bio-Sample numbers were PRJNA804266, SRR17927967, and SAMN25721206, respectively.
